# Molecular Mechanisms of Dual Potato Reproduction

**DOI:** 10.1111/ppl.70451

**Published:** 2025-08-18

**Authors:** Ania Kasprzewska, Aneta Basińska‐Barczak

**Affiliations:** ^1^ Department of Regulation of Gene Expression Institute of Plant Genetics of the Polish Academy of Sciences Poznań Poland; ^2^ Department of Integrative Plant Biology Institute of Plant Genetics of the Polish Academy of Sciences Poznań Poland

**Keywords:** flowering, potato, propagation, tuberisation

## Abstract

Changing environmental conditions caused by global warming threaten potato cultivation due to its sensitivity to both heat and drought. Unfortunately, classic potato breeding is slow to generate varieties that, whereas maintaining good yields, are resistant to unfavourable climatic conditions. Therefore, attempts are underway to create new potato varieties that can be grown from true seeds. However, this requires knowledge of flowering regulation in potatoes, because many cultivars that could be valuable parental lines do not flower. Although the molecular mechanisms regulating tuberisation are well‐characterised, our knowledge concerning flowering in potato is scarce. In this review, based on existing data for *Solanaceae* plants, we propose that the CONSTANS‐mediated photoperiodic regulatory pathway has been hijacked and adapted by the tuberisation process. We also indicate that potato plants have acquired alternative endogenous pathways related to plant ageing, sucrose availability or gibberellin signalling to induce flowering. We anticipate that, upon further experimental verification, the proposed mechanism may contribute to future progress in potato breeding and propagation.

## 1 Introduction

The world has changed. The average temperature on our globe has increased by 1.09°C over the past 120 years (Lee et al. 2023). Global warming has been accompanied by other changes, like seasonal droughts and aridification of agricultural lands. The pace of these changes has meant that existing cultivars are insufficiently adapted to current climatic conditions, whereas the human population is growing and the demand for food is increasing (Tilman et al. 2011). Thanks to its nutritional values, high level of minerals and vitamins (in particular vitamin C), as well as a satisfactory content of high‐quality proteins, potato is gaining popularity as an important food crop species (Burgos et al. 2020). According to the Food and Agriculture Organization Corporate Statistical Database (FAOSTAT) in 2022, the net potato tuber yield reached approximately 375 million tonnes, giving it the fourth place after corn, wheat and rice (https://www.fao.org/faostat/en/#home). Potato plants, however, are sensitive to high temperatures (Hancock et al. 2014; Hastilestari et al. 2018; Lehretz et al. 2019; Park et al. 2022) and water deficit (Cabello et al. 2012; Lehretz, Sonnewald, Lugassi, et al. 2021; Ramírez Gonzales et al. 2021), and respond to these adverse conditions with significant yield loss. It is predicted that, due to climate change, by 2040 potato tuber yield will decline by 32% (Hijmans 2003). This creates a high demand for new varieties that can give satisfactory yields in suboptimal environmental conditions. Almost all existing cultivars are vegetatively propagated, which frequently leads to pathogen accumulation, is expensive, and has a high carbon footprint (Zhang et al. 2021). For this reason, there is a growing number of research institutions pursuing the development of new potato varieties that can be multiplied by seeds (Jansky et al. 2016; Zhang et al. 2021). True potato seeds (TPS) are typically disease‐free, and their transport is significantly cheaper than seed tubers. Development of such varieties, however, poses numerous difficulties; not only because of the high heterozygosity of potato cultivars, but also because many of them have limited flowering and seed setting capacity (Jansky and Spooner 2018; Plantenga 2019). According to The European Cultivated Potato Database, approximately 35% of varieties for which data on flowering frequency are collected bloom occasionally or do not bloom at all. Such varieties cannot be used as parental lines, and their genetic material is not available for breeding programs.

Potato ( *Solanum tuberosum* L.) belongs to section *Petota* within the *Solanum* genus of the *Solanaceae* family. This species can be classified into two cultivar groups: *S. tuberosum* ssp. *andigena* (Andigena) composed of upland Andean varieties, and *S. tuberosum* ssp. *tuberosum* (Tuberosum) originated from Chilean landraces and comprising modern cultivars (Plantenga 2019; Prat 2010; Zierer et al. 2021). The tuberisation process in Andigena is under tight photoperiodic regulation. These species require a short day for tuber formation. On the other hand, Tuberosum produces tubers independently of the photoperiod, and short days only accelerate this process (Abelenda et al. 2014; Plantenga 2019). Tuberisation can also be influenced by temperature, light intensity, and nitrogen availability (Jackson 1999; Koch et al. 2024). Although mechanisms regulating tuberisation in potato have been well understood and described (Bao et al. 2025; Prat 2010; Rodriguez‐Falcon et al. 2006; Zierer et al. 2021), our knowledge of flowering mechanisms in this species is scarce. There is an ongoing debate regarding whether potato can be classified as a long‐day, short‐day or day‐neutral flowering plant (Almekinders and Struik 1996; González‐Schain et al. 2012; Plantenga 2019; Seibert et al. 2019). This is why it is so important to gather and systematise not only knowledge on tuberisation, but also on the flowering process in this species. In this work, we summarise the current state of knowledge; and based on the analysis of the existing experimental evidence, we present the key hypotheses and directions for future studies.

## 2 Various Roles of PEBP Family Proteins in Plant Reproduction

In Angiosperms, floral transition is under the control of the conserved PHOSPHATIDYLETHANOLAMINE‐BINDING PROTEINS (PEBP/CETS—CENTRORADIALIS, TERMINAL FLOWER 1, SELF PRUNING) gene family members. Based on sequence similarity to Arabidopsis paralogues, members of the PEBP family can be divided into 3 clades: FLOWERING LOCUS T‐like (FT‐like) clade coding for florigens, TERMINAL FLOWER 1/CENTRORADIALIS‐like (TFL/CEN‐like) coding for anti‐florigens, and MOTHER OF FT‐like (MFT‐like) coding for proteins regulating seed germination (Moreira et al. 2022; Zierer et al. 2021). Apart from flowering, proteins from this family are involved in the control of other developmental events, such as the formation of storage organs (bulbs, tubers, storage roots), regulation of shoot branching and root architecture, and seed germination (Jin et al. 2021; Moreira et al. 2022; Susila and Purwestri 2023). For example, in tomato ( *Solanum lycopersicum* ), the closest domesticated relative of potato, the ratio between florigenic SINGLE FLOWER TRUSS (SFT) and anti‐florigenic SELF PRUNING (SP) controls the repetitive vegetative‐reproductive switch and overall plant architecture (Carmel‐Goren et al. 2003; Jin et al. 2021; Shalit et al. 2009). In potato, the protein with anti‐florigenic activity is unknown. The possible candidate is TERMINAL FLOWER 1/CENTRORADIALIS (StCEN) protein since its overexpression represses flowering and tuberisation (Zhang et al. 2020). However, StCEN also represses, in a dose‐dependent manner, tuber sprouting, which is contradictory to the growth promotion by anti‐florigen (Morris, Alamar, et al. 2019). Besides StCEN, the potato PEBP family also includes tuberigen SELF PRUNING 6A (StSP6A) and its paralogs SELF PRUNING 3D (StSP3D), FLOWERING LOCUS T‐LIKE 1 (StFTL1), and SELF PRUNING 5G (StSP5G). Compared to Arabidopsis, which encodes only 6 *PEBP* genes, in the *Solanum* lineage, there has been a significant expansion of this family (Tomato Genome Consortium 2012). The potato genome contains 15 homologous genes encoding PEBP proteins (Table 1) (Abelenda et al. 2014). It remains an open question whether all of them are involved in tuberisation and to what extent they influence this process.

**TABLE 1:** *PEBP* genes in potato genome, their tomato orthologues and developmental function.

Potato PEBP gene	Function in potato development	Tomato ortholog	Function in tomato development
Sotub11g010050 StFTL1	Flowering and tuberisation inducer under SD (Jing et al. 2023)	Solyc11g008650 SP5G‐like (SP5G3/FTL1)	Flowering inducer in *S. pimpinellifolium* under SD (Song et al. 2020)
		Solyc11g008660 SP5G‐like (SP5G1/FTL1)	Not expressed (Cao et al. 2016)
	
Sotub11g010060 SP5G‐like	Unknown	Solyc11g008640 SP5G‐like (SP5G2)	Flowering repressor under SD (Cao et al. 2016)
Sotub05g026730 SP5G‐A	Tuberisation and flowering repressor under LD (Abelenda et al. 2016)	Solyc05g053850 SP5G	Flowering repressor under LD (Cao et al. 2016); (Song et al. 2020)
Sotub05g026750 SP5G‐B	Unknown	Solyc05g053850 SP5G	Flowering repressor under LD (Cao et al. 2016); (Song et al. 2020)
Sotub05g028860 SP6A	Tuberigen (Navarro et al. 2011)	Solyc05g055660 SP6A	Not expressed (Cao et al. 2016)
Sotub03g010860 SP3D	Florigen (Navarro et al. 2011), flowering and tuberisation inducer under SD (Jing et al. 2023)	Solyc03g063100 SFT/SP3D	Florigen (Shalit et al. 2009)
Sotub09g010600 SP9D	Unknown	Solyc09g009560 SP9D	Anit‐florigen, promotes vegetative growth (Fridman et al. 2002)
Sotub06g031280 SP/TFL1	Unknown	Solyc06g074350 SP/TFL1	Anit‐florigen, promotes growth and repress flowering (Shalit et al. 2009)
Sotub03g014740 StCEN/TERMINAL FLOWER‐1	Tuberisation and flowering repressor (Zhang et al. 2020), sprout growth repressor (Morris, Alamar, et al. 2019)	Solyc03g026050 CEN 1.1/SP3C	Flowering and germination repressor, modulates root architecture (Moreira et al. 2022)
Sotub01g016170 CEN 1	Unknown	Solyc01g009580 CEN 1.2	Unknown
		Solyc01g009560 CEN 1.3	
Sotub01g016150 CEN 1	Unknown	Solyc01g009580 CEN 1.2	Unknown
		Solyc01g009560 CEN 1.3	
Sotub01g016190 CEN 1	Unknown	Solyc01g009580 CEN 1.2	Unknown
		Solyc01g009560 CEN 1.3	
Sotub01g016180 CEN 1	Unknown	Solyc01g009580 CEN 1.2	Unknown
		Solyc01g009560 CEN 1.3	
Sotub03g032570 MFT	Unknown	Solyc03g119100 MFT/SP2I	Unknown
Sotub02g022270 SP2G	Unknown	Solyc02g079290 SP2G	Unknown

*Note:* Abelenda et al. (2016); Cao et al. (2016); Fridman et al. (2002); Jing et al. (2023); Moreira et al. (2022); Morris, Alamar, et al. (2019); Navarro et al. (2011); Shalit et al. (2009); Song et al. (2020); Zhang et al. (2020).

## 3 Molecular Mechanisms Regulating Potato Tuberisation

**FIGURE 1:**
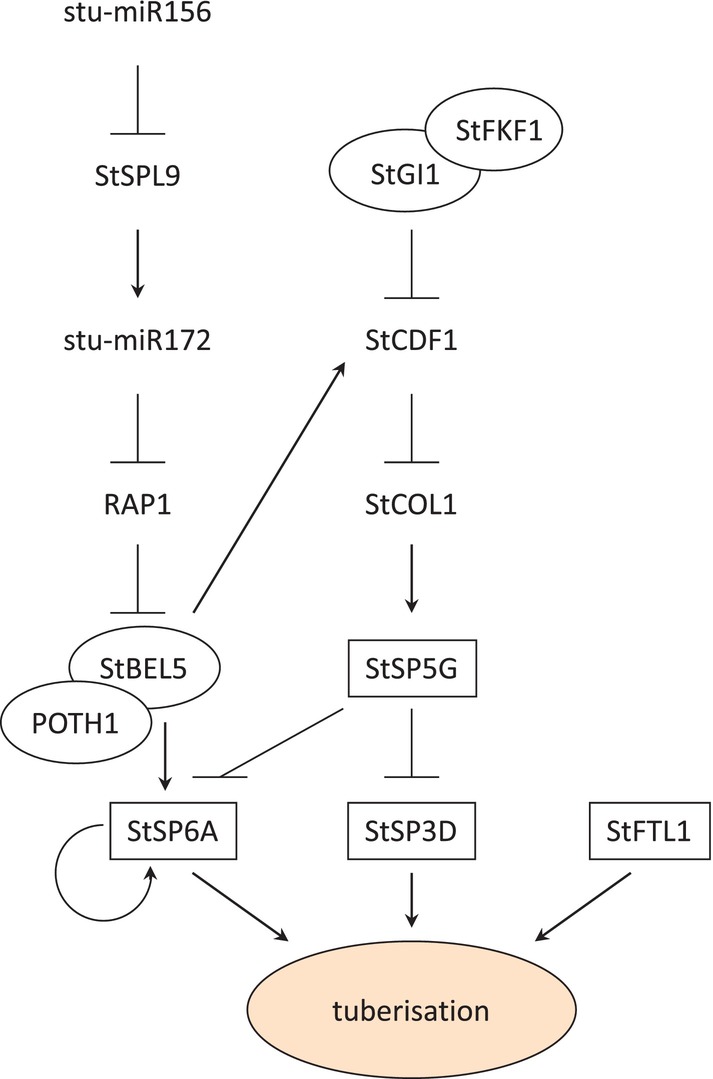
Simplified flowchart picturing two major pathways involved in tuberisation control in potato. Proteins forming functional complexes are shown in an ellipse. The PEBP family members are depicted as rectangles.

The tuberisation signal—tuberigen—is produced in leaves and transported via vascular tissue to stolons, where it induces tuber development (Bao et al. 2025; Prat 2010; Rodriguez‐Falcon et al. 2006; Zierer et al. 2021). Grafting experiments, in which flowering induced tobacco plants were grafted on uninduced potato rootstocks, showed that tuberigen can easily be substituted by the flowering signal—florigen (Chailakhyan et al. 1981). In potato, StSP6A, an ortholog of Arabidopsis FLOWERING LOCUS T (FT), is considered to be a tuberigen (Navarro et al. 2011). The tomato genome also encodes a gene paralogous to *StSP6A*. In wild tomatoes, *SP6A* is transcriptionally active, but in modern cultivars, it is inactive due to a premature stop codon (Carmel‐Goren et al. 2003; Navarro et al. 2011). The function of the *SP6A* gene in wild tomatoes is unknown. The transcript level of *StSP6A* in Andigena increases during short days, whereas in photoperiod‐neutral Tuberosum genotypes it correlates with the onset of the tuberisation process. Overexpression of *StSP6A* triggers tuberisation under long days in Andigena genotypes, whereas its silencing significantly delays this process under short‐day conditions (Navarro et al. 2011). The *StSP6A* expression level is under the control of at least two pathways: the miR156‐miR172 pathway operating through BELLRINGER‐1 like 5 (StBEL5), and the CYCLIN DOF FACTOR 1 (StCDF1)‐CONSTANS like 1 (StCOL1) pathway operating through StSP5G (Figure 1). Both pathways are responsive to photoperiod stimuli, but there is evidence that miR156‐miR172 could also be regulated by sucrose availability (Garg et al. 2021).

### 3.1 Tuberisation Regulation by miR156‐miR172 Pathway

**FIGURE 2:**
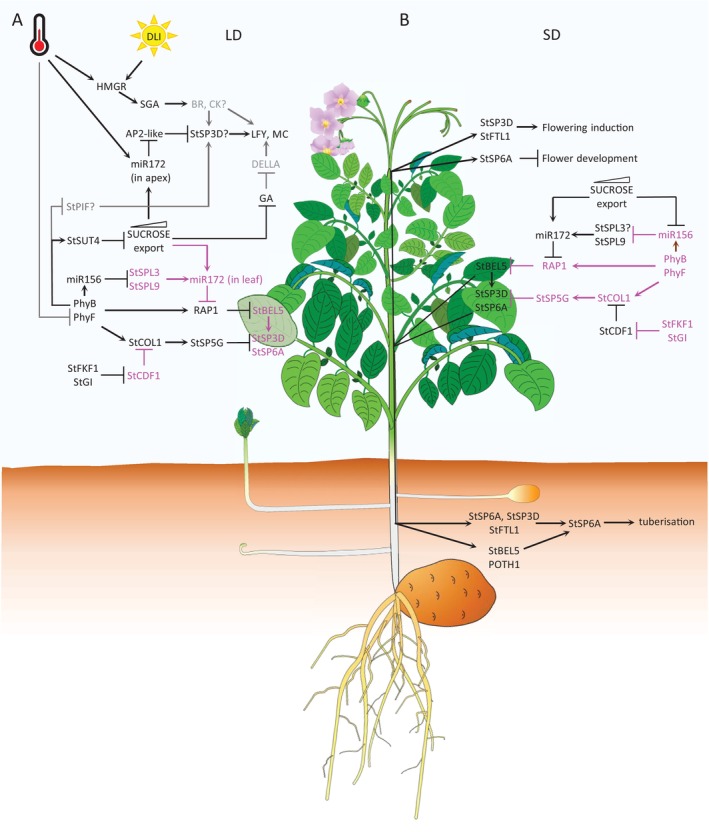
Interplay between mechanisms regulating tuberisation and flowering in potato. (A) Potato plant growing under long‐day conditions (LD). Active PhyB and PhyF stabilise stu‐miR156, RAP1, and StCOL1 in potato leaves. Stu‐miR156 targets *StSPL* for degradation, and all tuberisation signals (StSP6A, StBEL5, and stu‐miR172) are repressed. Despite this, both levels and export of sucrose gradually increase. In the shoot apex, increasing sucrose levels repress GA biosynthesis and induce stu‐miR172, which targets the AP2‐like family flowering repressors. High Day Light Integral (DLI) and high temperature induce flowering through 3‐hydroxy‐3‐methylglutaryl coenzyme A reductase (HMGR) and stimulation of steroid glycoalkaloids (SGA) production. (B) Potato plants growing under short‐day (SD) conditions. All tuberisation repressors (stu‐miR156, StSP5G, and RAP1) are downregulated. Tuberisation‐inducing molecules are synthesised and subsequently delivered through the phloem to stolons and the shoot apex. In stolons, StBEL5 and StSP6A induce StSP6A expression, which leads to tuberisation. In the shoot apex, StSP3D induces flowering, but StSP6A inhibits flower development. Molecules and pathways engaged in particular steps are depicted in black, whereas those not involved or switched off are indicated in magenta. Proposed interactions are in grey.

MicroRNAs (miRNAs) are small RNA molecules encoded within the genome and processed by endonucleases from hairpin precursors. They perform regulatory functions by directing targeted transcripts for degradation or for translational inhibition (Axtell and Meyers 2018). In Arabidopsis, the regulatory microRNA ath‐miR156 is responsible for both juvenile to adult and vegetative to generative phase transitions (Hyun et al. 2016). It is present in high amounts in young leaves and decreases with organ age progression and with an increase of sucrose availability (Yang et al. 2013; Yu et al. 2013, 2015). In potato, the level of stu‐miR156 in leaves is influenced by age and also by photoperiod (Figure 2A) (Bhogale et al. 2014). Under long‐day conditions, stu‐miR156 accumulates to higher levels than in short days and targets transcripts of *SQUAMOSA PROMOTER BINDING‐like 9* (*StSPL9*) factor for degradation (Bhogale et al. 2014) leading to the lack of stu‐miR172 expression induction. In the absence of stu‐miR172, RELATED TO APETALA2 1 (RAP1) protein accumulates and represses *StBEL5* expression (Martin et al. 2009). RAP1 belongs to the AP2‐like family of flowering repressors and is involved in tuberisation inhibition (Martin et al. 2009). Under short‐day conditions, stu‐miR156 expression drops to a level that can no longer control the *StSPL9* (Figure 2B). This leads to the accumulation of StSPL9, which binds to the stu‐miR172 promoter and triggers its expression (Bhogale et al. 2014). In turn, stu‐miR172 degrades transcripts of *RAP1* and releases *StBEL5* gene transcription (Martin et al. 2009). StBEL5 belongs to the BEL1‐like transcription factor family, and its overexpression in potato leads to the induction of tuberisation under non‐inductive long‐day conditions (Chen et al. 2003). Its mRNA is synthesised in leaves and transported via phloem to stolons where, along with its partner POTATO HOMEOBOX 1 (POTH1), it regulates tuberisation by inducing the expression of genes degrading gibberellins (GAs) and synthesising cytokinins (CKs) and through local upregulation of *StSP6A* expression (Banerjee et al. 2006; Chen et al. 2003; Sharma et al. 2015). In addition, *StSP6A* expression in stolons is under the control of an autoregulatory loop (Navarro et al. 2011) (Figure 1).

### 3.2 Tuberisation Regulation by StCDF1‐StCOL1 Pathway

Photoperiodic pathway mediated through StCDF1‐StCOL1 represses tuber initiation under unfavourable conditions (Figure 2A). Under long days, FLAVIN‐BINDING, KELCH REPEAT, F‐BOX 1 (StFKF1) induced by blue light complexes with GIGANTEA (StGI1) and targets StCDF1 for proteolytic degradation (Kloosterman et al. 2013). Since StCDF1 is a repressor of *StCOL1*, an ortholog of Arabidopsis COMSTANS (CO), StCOL1 protein accumulates to higher levels and induces expression of *StSP5G*, considered to be a major repressor of tuberisation during long‐day photoperiods (Abelenda et al. 2016; Navarro et al. 2011). Under short‐day conditions favouring tuberisation, StCDF1 protein is no longer targeted for degradation, as the StGI1‐StFKF1 complex does not form, and represses the expression of *StCOL1*, releasing the inhibitory effect exerted by StSP5G on *StSP6A* (Figure 2B). StCDF1 protein plays a crucial role in the biology of potato cultivars. A loss of the StGI1‐StFKF1 binding site in naturally truncated alleles of *StCDF1* has enabled potato cultivars to tuberise independently from photoperiod (Kloosterman et al. 2013).

**FIGURE 3:**
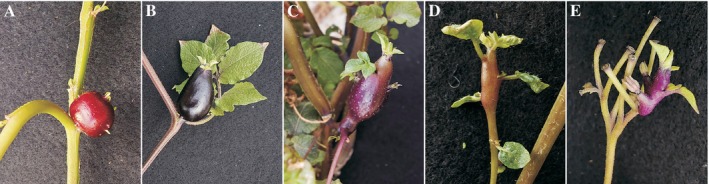
Aerial tubers developing on *Solanum tuberosum* ssp. *andigena* 7540 wild type plants. Plants were grown from seeds for 2 months in a growth chamber with 16 h of daylight; before being transferred to the greenhouse, where natural light and a short‐day photoperiod were maintained. (A) and (B) sessile tubers developing from an axillary bud, (C) and (D) aerial tubers developing from the stem of an axillary shoot, (E) aerial tuber developing within the inflorescence after flower shedding.

Despite that StBEL5 induces *StCDF1* and *StSP6A* expression (Sharma et al. 2015) it seems that StCDF1‐StCOL1 is the main pathway controlling tuberisation onset in response to photoperiod. The level of *StBEL5* is under miR156‐miR172 control, and plants overexpressing stu‐miR172 initiate tuberisation earlier than wild‐type forms (Martin et al. 2009). However, in these plants, the inhibiting effect of long days on tuberisation can be observed. This suggests that unfavourable photoperiod additionally suppresses tuberisation via another signalling pathway, perhaps by regulating StCOL1. Moreover, the StCDF‐StCOL1 pathway can bypass the repression of tuberisation caused by miR156 and juvenility of the plant, especially when environmental conditions strongly favour tuberisation. It was observed that some Tuberosum plants growing from TPS under short photoperiod can form tubers in one true leaf stage (Ewing 1995). Interestingly, the overexpression of stu‐miR156 in potato plants growing under a tuberisation‐inducing photoperiod decreased the yield of underground tubers and triggered the formation of aerial tubers (Bhogale et al. 2014). Aerial tubers are formed on the above‐ground shoots from the axillary buds, which should remain dormant during a typical tuberisation process (Ewing 1995). A similar phenotype has been observed in potato plants upon silencing of *BRANCHED1b* (*BRC1b*) transcription factor, and *StBMI1‐1* or overexpression of *MULTICOPY SUPPRESSOR OF IRA1* (*StMSI1*), and *StSWEET11* (Abelenda et al. 2019; Kumar et al. 2019; Nicolas et al. 2022). Mechanisms driving aerial tuber formation are based on epigenetics and sucrose partitioning (Abelenda et al. 2019; Kondhare et al. 2021; Kumar et al. 2019; Nicolas et al. 2022). Moreover, this phenotype is characteristic of plants with disturbed translocation of tuberisation stimuli and occurs naturally when plants are strongly induced for tuberisation (Ewing and Wareing 1978) (Figure 3). However, overexpression of stu‐miR156 did not influence the timing of tuberisation onset (Bhogale et al. 2014). This suggests that stu‐miR156 expression alone is not sufficient to completely prevent the generation of tuberisation signals under favourable conditions, so it seems that the transition from juvenile to the adult stage and *StBEL5* movement is not an indispensable condition for potato tuberisation. Although the promoter of *StSP6A* requires StBEL5 for activation (Sharma et al. 2015), it is possible that under short‐day conditions, other FT‐like genes are induced in a StCOL1‐dependent but StBEL5‐independent way. Recently, it was shown that under short‐day conditions, two paralogs of StSP6A, StSP3D and StFTL1, are synthesised in potato leaves and move via phloem to stolons, where they are able to induce tuberisation (Jing et al. 2023).

## 4 Molecular Mechanisms Regulating Flowering

In contrast to tuberisation regulation, our knowledge of mechanisms controlling flowering in potato is still limited. Although in Tuberosum genotypes, flowering and tuberisation usually occur together at the same time, in Andigena, these two processes can be separated by manipulating the length of the day, since tuberisation takes place strictly in short days. But when it comes to flowering, we cannot even be sure if Andigena potato can be classified as a long‐, short‐day, or a day‐neutral plant (Almekinders and Struik 1996; González‐Schain et al. 2012; Plantenga 2019; Seibert et al. 2019).

### 4.1 Potato Flowering Regulation by Photoperiod

The experimental data regarding flowering regulation by photoperiod in potato are ambiguous and contradictory. For example, detailed analysis of results presented by Martin et al. (2009) shows that Andigena flowers earlier in long days (after 42 days versus 53 days in SD), yet simultaneously has more leaves (22 leaves under LD condition and 20 leaves under SD) than plants growing in short days, what may indicate that the vegetative stage is prolonged in longer photoperiod. Seibert et al. (2019) also found that Andigena plants flower significantly earlier in LD photoperiod, but they did not find any correlation between total leaf number and shoot apical meristem transition to flowering. On the other hand, Plantenga, Heuvelink, et al. (2019) in the most detailed experimental analysis of the influence of light conditions on potato flowering time found that plants flower after the same number of days regardless of photoperiod, but they have more leaves under long days. Moreover, they found that increasing the amount of light perceived by plants growing in short‐day conditions reduces the number of days needed for flowering (Plantenga 2019; Plantenga, Bergonzi, et al. 2019). However, short days induce tuberisation, which causes repression of flowering due to frequent flower abortion (Figure 3E) (Ewing 1995; Plantenga 2019; Plantenga et al. 2018). It seems that StSP6A inhibits flower development (Plantenga et al. 2018) and can interact with StSWEET11, blocking its activity and setting up strong sink formation in developing tubers (Abelenda et al. 2019; Lehretz, Sonnewald, and Sonnewald 2021).

In potato, StSP3D is considered to be the florigen (Navarro et al. 2011). Silencing of *StSP3D* results in delayed flowering (Navarro et al. 2011; Teo et al. 2016), whereas overexpression not only accelerates flowering but also additionally induces the transition of the stolon apical meristem into the flower meristem (Jing et al. 2023). A similar meristem transition was observed in potato plants overexpressing *Hd3a*, the rice ortholog of *FT* (Navarro et al. 2011), and in the case of interspecific hybrids between *S. tuberosum* ssp. *tuberosum* and *S. etuberosum* , containing a higher proportion of tuberosum genomes (Ehlenfeldt and Hanneman 1984). Under long‐day conditions, expression of *StSP3D* in potato leaves is very weak or undetectable (Navarro et al. 2011; Teo et al. 2016). Short days and high light, in particular a high daylight integral (DLI), upregulates *StSP3D* expression (Jing et al. 2023; Navarro et al. 2011; Plantenga, Bergonzi, et al. 2019; Teo et al. 2016). *StSP3D* levels are negatively impacted by StCOL1 (Abelenda et al. 2016). Most probably, this is an indirect effect of the induction of *StSP5G* expression by StCOL1 (Figure 2A). StSP5G is likely to be the repressor of StSP3D. Both types of plants, those with silenced *StCOL1* and those with silenced *StSP5G*, display accelerated flowering (Abelenda et al. 2016). In tomato, a similar mechanism of photoperiod‐related flowering regulation exists (Soyk et al. 2017). Long days trigger an accumulation of SlSP5G, which prevents flowering via repression of *SlSP3D/SINGLE FLOWER TRUSS* (*SlSFT*) gene expression. Natural variations in *SlSP5G* promoter‐enhancer interaction have freed tomato flowering from the inhibiting effect of long‐day photoperiods (Zhang et al. 2018). The SlSP5G2, a paralog of SlSP5G, represses tomato flowering under short days (Cao et al. 2016). Additionally, naturally occurring *SlSP5G1/SlSP5G3/Flowering Locus T‐Like 1* (*SlFTL1*) alleles, responsible for flowering stimulation via induction of *SlSP3D/SlSFT* expression under short days (Table 1), caused tomato transformation from a short‐day to a day‐neutral plant (Song et al. 2020).

Despite the expression of the flowering repressor gene *StSP5G* being controlled by StCOL1, so far, there is no unequivocal evidence on the involvement of CONSTANS in potato plant flowering regulation. Some reports showed that the overexpression of *AtCO* in the Andigena genotype did not affect flowering time (Martínez‐García et al. 2002), whereas others report day‐length independent flowering delays (González‐Schain and Suárez‐López 2008). When it comes to *StCOL1*, a photoperiod‐independent effect of flowering acceleration has been observed for both overexpression and silencing of this gene (Abelenda et al. 2016; González‐Schain et al. 2012). Interestingly, overexpression of the gain‐of‐function allele of the *StCDF1.2* gene (a negative regulator of *StCOL1*) either in Andigena or Tuberosum does not affect flowering time, whereas in Arabidopsis the onset of this process is delayed (Kloosterman et al. 2013). This may indicate that the CDF‐CONSTANS mediated pathway is not as essential in potato flowering as it is in potato tuberisation and Arabidopsis flowering regulation. In Arabidopsis, both CO and CDF1 are regulated by blue light, which has a positive impact on flowering (Valverde et al. 2004). In potato, however, no influence of blue or far‐red light on flowering was observed (Plantenga et al. 2016).

### 4.2 Potato Flowering Regulation by Phytochromes (Light Quality and Temperature)

Phytochromes are responsible for the perception of red and far‐red light. There are five phytochrome proteins encoded in the potato genome: StPHYA, StPHYB, StPHYB2, StPHYE and StPHYF (Zhou et al. 2019). StPHYA is involved in the synchronisation of the circadian clock (Yanovsky et al. 2000), whereas StPHYB and StPHYF control tuberisation onset in response to photoperiod (Jackson 1999; Zhou et al. 2019). Moreover, StPHYF represses flowering, since potato plants with silenced *StPHYF* display accelerated flowering (Wang et al. 2023). The mechanism of *StSP3D* repression by StPHYF is not known. It is probable that StPHYF represses flowering acting similarly to StPHYB by stabilisation of StCOL1, and triggering this way expression of *StSP5G*, which represses expression of *StSP3D* in response to photoperiod (Figure 2A) (Wang et al. 2023; Zhou et al. 2019). However, taking into account the probable photoperiod‐independent flowering regulation in potato, it is possible that StPHYF influences flowering in an StCOL1‐independent manner, perhaps through targeting of PHYTOCHROME INTERACTING PROTEINS (PIFs) for degradation, as it happens in Arabidopsis (Balcerowicz 2020; Freytes et al. 2021). It was shown that in this plant, PIF4, PIF5 and PIF7 can directly induce florigen expression in response to changes in red to far red ratio (Galvāo et al. 2019). Seven genes encoding PIF members have been identified in the potato genome, and at least *StPIF3* is involved in the regulation of shading avoidance syndrome (Han et al. 2024). However, it is not known if *StPIF3* influences potato flowering.

In Arabidopsis, both PHYB and PIFs can also mediate information about temperature (Balcerowicz 2020; Galvāo et al. 2019; Jung et al. 2016; Legris et al. 2016). It seems equally likely that StPHYF does not convey information on the day length or quality of light, but rather is involved in temperature signalling. It has been shown that StPHYF heterodimerises with StPHYB (Zhou et al. 2019). Perhaps in potato, in which elevated temperature stops tuberisation and promotes flowering (Lehretz, Sonnewald, and Sonnewald 2021), it is temperature that has a stronger impact on flowering time than photoperiod or light quality.

### 4.3 Potato Flowering Regulation by High Ambient Temperature

Different groups found that a higher ambient temperature represses tuberisation but promotes flowering (Almekinders and Wiersema 1991; Almekinders and Struik 1996; Lehretz, Sonnewald, and Sonnewald 2021). Moderate heat may influence flowering through transcriptional and posttranscriptional down‐regulation of *StSP6A*, a repressor of flower development (Hancock et al. 2014; Lehretz et al. 2019; Morris, Ducreux, et al. 2019; Park et al. 2022; Plantenga 2019; Plantenga et al. 2018). Moreover, high temperature causes a redirection of assimilates from tubers to leaves, supporting shoot development (Hastilestari et al. 2018). Transcriptomic data obtained for potato growing under heat stress conditions also revealed that high temperature decreased the expression level of *StPHYB2* (Hastilestari et al. 2018). It is possible that StPHYB2, similarly to StPHYF, is involved in controlling potato flowering, but this hypothesis needs further elucidation.

Ambient temperature may also influence flowering by modulating miR156 and miR172 levels. At 16°C, Arabidopsis accrues ath‐miR156, whereas 23°C promotes ath‐miR172 accumulation (Lee et al. 2010).

### 4.4 Age‐Dependent Flowering Regulation in Potato—miR156‐miR172 Pathway

After sprouting, plants do not immediately possess the ability to propagate. They are in the vegetative juvenile stage, and in order to produce seeds, they must acquire competence to flower (Hyun et al. 2016). In Arabidopsis, the regulatory microRNA ath‐miR156 is responsible for the maintenance of a plant both in its juvenile stage and in its vegetative stage (Hyun et al. 2016; Wu and Poethig 2006). Ath‐miR156 targets for degradation transcripts of AtSPL3 and AtSPL9, which would otherwise directly activate the expression of flower identity genes like *FT*, *FRUITFUL* (*FUL*), *LEAFY* (*LFY*), *SUPPRESSOR OF OVEREXPRESSION OF CONSTANS 1* (*SOC1*), and *APETALA 1* (*AP1*) leading to the development of floral organs (Kim et al. 2012; Wang et al. 2009; Yamaguchi et al. 2009). In potato, *StSPL3* and *StSPL9* gene transcripts are also targets of stu‐miR156, and overexpression of this miRNA results in a lack of flowering (Bhogale et al. 2014). It has been shown that StSPL9 is involved in the regulation of tuberisation through binding to the stu‐miR172 promoter and triggering its expression (Bhogale et al. 2014). The role of StSPL3 remains elusive. It would be worth testing whether StSPL3 can directly activate floral identity genes in potato, as it happens in Arabidopsis.

A gradual decrease in miR156 expression and miR172 induction accompanies the transition from a vegetative to a reproductive state. The miR172 expression marks the vegetative‐reproductive developmental phase change. Once miR172 is produced, the plant is readied for propagation. In Arabidopsis, ath‐miR172 targets the *AP2‐like* transcription factors involved in flowering repression. Potato plants overexpressing stu‐miR172 initiate flowering earlier than wild‐type forms (Martin et al. 2009). Thus, stu‐miR172 may regulate flowering in potato, targeting transcripts homologous to Arabidopsis *AP2* and *TARGET OF EAT1* (*TOE1*). So far, it has only been shown that potato stu‐miR172 degrades *RAP1*, which belongs to the AP2‐like family of flowering repressors involved in tuberisation inhibition (Martin et al. 2009).

Interestingly, the overexpression of stu‐miR156 or stu‐miR172 in potato has a stronger impact on flowering than on tuberisation (Bhogale et al. 2014; Martin et al. 2009). Although overexpression of stu‐miR156 does not affect the timing of tuberisation signal formation, it completely blocks flowering (Bhogale et al. 2014). On the other hand, overexpression of stu‐miR172 causes potato plants to flower earlier regardless of photoperiod (Martin et al. 2009). It seems that in potato, the miR156‐miR172 pathway controls the acquisition of competence to flower as it happens in other flowering plants.

The miR156‐miR172 pathway may be under the control of sugar availability (Hyun et al. 2016). In Arabidopsis, the application of sucrose downregulates ath‐miR156 expression, whereas in potato, it upregulates stu‐miR172 level (Garg et al. 2021).

### 4.5 Potato Flowering Regulation by Sucrose and Gibberellins

Potato plants with silenced *SUCROSE TRANSPORTER 4* (*StSUT4*) display phenotypes resembling stu‐miR172 overexpressors (Garg et al. 2021). *StSUT4*‐RNAi plants have shortened internodes, disrupted shade avoidance syndrome, and are insensitive to red light (Chincinska et al. 2008; Liang et al. 2023). Moreover, they have enhanced sucrose efflux and higher content of soluble sugars in the shoot apex, leading to up‐regulation of stu‐miR172 expression and acceleration of tuberisation and flowering (Chincinska et al. 2008; Garg et al. 2021). It has been postulated that this phenotype could be related to hampered gibberellic acids (GA) biosynthesis, due to down‐regulation of *StGA20ox1* transcript (Chincinska et al. 2008). The phenotype of *StSUT4*‐silenced potato plants resembles *SlSUT4* silencing in tomato (Liang et al. 2023), which resulted in a SlMYB76‐mediated downregulation of *SlGA20ox1* expression. Interestingly, expression of *PROCERA/DELLA* and floral meristem identity genes *LFY/FALSIFLORA* (*FA*) and *AP1/MACROCALYX* (*MC*) was elevated in these lines, whereas expression of floral integrator genes *SOC1* and *SFT/SlSP3D* was not altered. The phenotypic similarity between *StSUT4*‐RNAi and *SlSUT4*‐RNAi plants suggests that, in potato, as in tomato, sucrose may induce flowering without the accumulation of florigen, whereas GA can repress flowering via DELLA proteins (Figure 2A) (Liang et al. 2023). Treatment of potato plants with GA solution has an inhibitory effect on flower development (Chincinska et al. 2008). Similarly, exogenous application of GA on potato plants having delayed flowering caused by overexpression of *AtCO* did not restore the wild‐type phenotype (González‐Schain and Suárez‐López 2008).

In Arabidopsis, where GAs stimulate flowering, the delay of this process during insufficient doses of light is mediated by MYB‐driven transcriptional repression of the GA biosynthesis pathway (Zhao et al. 2011). It is a matter of question if potato responses to high light intensity are also mediated via GA synthesis or response pathways.

### 4.6 Potato Flowering Regulation by High Day Light Integral

The majority of reports on flowering in potato agreed upon the fact that high light intensity or high DLI induces this process (Plantenga 2019). It seems, however, that a high DLI induces flowering independently from florigen upregulation because it accelerates this process also in plants with silenced *StSP3D* expression (Plantenga, Bergonzi, et al. 2019). So far, there is not enough data to explain the flowering induction mechanism governed by high light intensity in potato. This process could be mediated equally by sucrose and GAs, or by steroid glycoalkaloids (SGAs). High light intensity and high temperature can enhance SGA production in potato (Ginzberg et al. 2012). The key enzyme catalysing the first step of isoprenoid biosynthesis, necessary for SGA production, is 3‐hydroxy‐3‐methylglutaryl coenzyme A reductase (HMGR) (Ginzberg et al. 2012; Moehninsi et al. 2020). The presence of active homologues encoding this enzyme in potato floral tissues suggests their involvement in flower development (Korth et al. 1997). Overexpression of *StHMGR1*, involved in the mevalonic acid (MVA) pathway of isoprenoid biosynthesis, led to earlier flowering in potato, whereas overexpression of *StHMGR3*, which functions in the methylerythritol phosphate (MEP) pathway, did not influence this process (Moehninsi et al. 2020). Amongst other aspects, the MVA pathway is linked to CKs and brassinosteroid (BR) biosynthesis, whereas MEP is involved in GA and ABA production (Liao et al. 2016; Vranová et al. 2013). This points out the possible involvement of CKs and BRs in the regulation of the flowering process in potato (Figure 2A).

## 5 Conclusion

In the South American Andes, the primary domestication region of potato, these plants do not face major photoperiodic variation. The difference between the longest day and the shortest day is less than an hour. Such limited variation also applies to the annual temperature distribution in this region. The potato tuberisation process employs molecular machinery responsible for flowering regulation in other species. Under long‐day conditions, StCOL1 induces the expression of *StSP5G*, the repressor of both tuberigen (StSP6A) and florigen (StSP3D). Under short‐day conditions, StCDF1 turns off the expression of *StCOL1*, which enables the induction of both *StSP6A* and *StSP3D* gene expression; however, the tuberisation signal represses flower development. Despite this repression, potato has the capacity to launch the flowering process; Andigena can initiate flowering before tuberisation, whereas in Tuberosum, a mutation in the *StCDF1* gene has led to *StCOL1* downregulation and equalised the timing of these two processes, so they can overlap. Thus, it is conceivable that under long‐day conditions, flowering regulation in potato may be governed by pathways other than photoperiod. There is a strong possibility that potato flowering is age‐regulated (regulated by the miR156/miR172 ratio), sucrose‐, or GA‐dependent. All of these pathways may interact to ensure flowering initiation at the most beneficial time for potato plants. As the summer passes, days are shortened, and the temperature drops, and the tuberisation path is initiated. The abovementioned hypothetical scenarios still await further experimental confirmation; however, we believe that this direction may bring breakthroughs in our understanding of the flowering process in potato and revolutionise its generative propagation.

## Author Contributions

A.K. prepared the concept of this review, wrote the first draft of the manuscript, and, together with A.B.B., worked on the final version. A.B.B. was involved in the conceptualization of the graphical part of the manuscript and the technical aspects of figure preparation.

## Conflicts of Interest

The authors declare no conflicts of interest.

## Data Availability

The authors have nothing to report.
